# 

**Published:** 1846-03

**Authors:** 


Page

### LIBRARY.

A Treatise on Second Dentition, and the Natural Method of directing it; followed by a Summary of Stomatic Semeology. Translated from the French of C. F. Delabarre, by *-A-*........... 29

### JOURNAL.

Article I.—An Address, delivered at the opening of the Dental College of Ohio, at request of Faculty, by Rev. B. P. Aydelott, D. D.,........ 185

Article II.—Opening Address. Delivered before the Pennsylvania Association of Dental Surgeons, by Robert Arthur, D. D. S.,........... 195

Article III.—Transactions of the Pennsylvania Association of Surgeon Dentists,............................................... 208

Article IV.—An Essay on the Origin, Development, and Eruption of the Teeth, by C. O. Cone, D. D. S., &c.,........................ 221

Article V.—Observations on Artificial Obturators and Palates, by B. B. Brown, M. D., D. D. S.,.................................... 232

Article VI.—Letter from C. C. Allen, Dentist, of New York,......... 246

Article VII.—Correspondence,.................................. 252

### COLLECTANEA.

Calculi of the Nasal Fossæ, by M. Demarquay,...................... 254

Death from Aconite,............................................ 255

Claims of the Temperance Reformation,.......................... 256

Materia Medica in Rhyme,....................................... 256

### BIBLIOGRAPHICAL NOTICES.

The Natural History and Diseases of the Human Teeth, by Joseph Fox, M. R. C. S. L., &c. Remodeled, with an Introduction and numerous additions, by Chapin A. Harris, M. D., D. D. S., &c.,............ 257

Introductory Lectures in the Medical Department of Pennsylvania College, 258

Lectures on the Operations of Surgery, &c. &c. By Robert Liston, Esq., &c. 258

Principles of Pathological Anatomy. By J. Hope, M. D., F. R. S., &c.,.. 258

The American Journal of Insanity; and, Report of the Pennsylvania Hospital for the Insane,......................................... 259

The Half-Yearly Abstract of the Medical Sciences,................... 259

The Southern Journal of Medicine and Pharmacy,.................... 259

The Anatomical Remembrancer, or Complete Pocket Anatomist, &c. &c.,. 259

Preservation of Health. By Jno. C. Warren, M. D.,................... 260

Elements of Pathological Anatomy. By Sam'l D. Gross, M. D.,......... 260

Outlines of the Arteries and Nerves. By John Neill, M. D.,........... 260

### MISCELLANEOUS NOTICES.

Baltimore College of Dental Surgery,.............................. 261

Medico-Dental Education,......................................... 261

Editorship of the Journal,........................................ 263

Report of Committee appointed to visit Members of A. S. D. S., in New York, 264

